# Subsequent maternal sleep deprivation aggravates neurobehavioral abnormalities, inflammation, and synaptic function in adult male mice exposed to prenatal inflammation

**DOI:** 10.3389/fnbeh.2023.1226300

**Published:** 2023-07-25

**Authors:** Yue-Ming Zhang, Meng-Ying Zhang, Ru-Meng Wei, Jing-Ya Zhang, Kai-Xuan Zhang, Bao-Ling Luo, Yi-Jun Ge, Xiao-Yi Kong, Xue-Yan Li, Gui-Hai Chen

**Affiliations:** ^1^Department of Neurology (Sleep Disorders), The Affiliated Chaohu Hospital of Anhui Medical University, Hefei, Anhui, China; ^2^Department of Anesthesiology, The Affiliated Chaohu Hospital of Anhui Medical University, Hefei, Anhui, China

**Keywords:** maternal sleep deprivation, lipopolysaccharide, inflammation, synaptic proteins, anxiety, depression, cognition

## Abstract

**Objective:**

Studies have suggested that prenatal exposure to inflammation increases the risk of neuropsychiatric disorders, including anxiety, depression, and cognitive dysfunction. Because of anatomical and hormonal alterations, pregnant women frequently experience sleep dysfunction, which can enhance the inflammatory response. The aim of this study was to explore the effects of maternal sleep deprivation on prenatal inflammation exposure-induced behavioral phenotypes in offspring and identify the associated mechanisms.

**Methods:**

Pregnant mice received an intraperitoneal injection of lipopolysaccharide (LPS) on gestational day 15 and were subsequently subjected to sleep deprivation during gestational days 15–21. Anxiety-like behavior was evaluated by the open field test and the elevated plus maze test. Depression-like behavior was assessed by the tail suspension test and the forced swimming test. Cognitive function was determined using the Morris water maze test. The levels of markers of inflammation and synaptic function were examined employing general molecular biological techniques.

**Results:**

The results showed that prenatal exposure to LPS resulted in anxiety- and depression-like symptoms and learning and memory deficits, and these effects were exacerbated by maternal sleep deprivation. Furthermore, maternal sleep deprivation aggravated the prenatal LPS exposure-induced increase in the expression of interleukin (IL)-1β, IL-6, and tumor necrosis factor-α and decrease in the levels of postsynaptic density-95 and synaptophysin in the hippocampus.

**Discussion:**

Collectively, these results suggested that maternal sleep deprivation exacerbates anxiety, depression, and cognitive impairment induced by prenatal LPS exposure, effects that were associated with an inflammatory response and synaptic dysfunction.

## 1. Introduction

Prenatal exposure to adverse factors results in changes in brain structure and developmental dysfunction in adulthood, leading to neuropsychiatric behavioral abnormalities (Knorr and Fox, 2023; Mansfield et al., 2023; Shimizu et al., 2023). Viral or bacterial infection during pregnancy may results in the fetus being exposed to an inflammatory environment *in utero*. Clinical studies have found that prenatal inflammation is associated with an increased risk of neurodevelopmental conditions, including autism spectrum disorder, attention-deficit/hyperactivity disorder, and Tourette’s syndrome (Han et al., 2021; Usui et al., 2023; Vázquez-González et al., 2023). Several preclinical studies have reported that prenatal exposure to inflammation induced by the intraperitoneal administration of lipopolysaccharide (LPS) can enhance anxiety, depression, and cognitive impairment in offspring (Paris et al., 2011; Zhuang et al., 2021).

Likely due to pregnancy-related anatomical, physiological, and hormonal changes, approximately half of pregnant women experience sleep dysfunction, including short sleep duration, poor sleep quality, frequent wakefulness, and difficulty falling asleep, which can adversely affect their offspring’s emotional and cognitive functions (Izci-Balserak et al., 2018; Sedov et al., 2018; Polo-Kantola, 2022). One study demonstrated that maternal sleep deprivation significantly enhanced anxiety, depression, and cognitive impairment in offspring (Peng et al., 2016). The offspring of damns exposed to sleep deprivation on gestational day 18 (GD18) showed hippocampus-dependent spatial learning and memory deficits in the Morris water maze test and depressive behavior in the sucrose preference test (Zhao et al., 2014). Additionally, growing evidence suggests that sleep is closely associated with immune function (Everson, 1993; Everson and Toth, 2000; Al-Abri et al., 2023). Severe sleep disorder reduces natural and cellular immunity by inducing alterations in the cytokine network (Irwin, 2002). Xu et al. (2020) showed that sleep deprivation further enhances the LPS-mediated upregulation of proinflammatory cytokine levels in plasma and the hippocampus of adult mice, suggesting that sleep deprivation exacerbates LPS-induced inflammation. In some underdeveloped regions of the world, infection and sleep dysfunction occur simultaneously in pregnant women (Pluciński et al., 2012). However, the effects of maternal sleep deprivation on prenatal inflammation exposure-induced neurobehavioral abnormalities in offspring mice are unclear.

Inflammation is known to be closely associated with anxiety, depression, and cognitive impairment. Maternal immune activation was shown to result in anxiety- and depression-like behaviors, cognitive impairment, and increased levels of proinflammatory cytokines, including interleukin (IL)-1β, IL-6, and tumor necrosis factor-alpha (TNF-α) in offspring (Rahimi et al., 2020; Zhang Z. et al., 2022). Additionally, maternal sleep deprivation led to microglial activation and increased the concentrations of proinflammatory cytokines in the hippocampus, which contributed to maternal sleep deprivation-induced cognitive dysfunction. Changes in synaptic plasticity may be another mechanism underlying anxiety, depression, and cognitive impairment. Postsynaptic density protein 95 (PSD-95) and synaptophysin (SYN) are important synaptic proteins localized to the postsynaptic and the presynaptic membranes, respectively. They regulate synaptic plasticity, synaptic vesicle release, and synaptic maturation, which are closely related to anxiety, depression, and cognitive impairment (Huang et al., 2021; Deng et al., 2022; Jia et al., 2023). Prenatal exposure to LPS significantly decreased SYN expression in offspring concomitant with spatial learning and memory impairments (Hao et al., 2010). We have previously shown that maternal sleep deprivation increases anxiety, depression, and cognitive dysfunction, which are associated with reductions in the expression of brain-derived neurotrophic factor (BDNF), PSD-95, and SYN in the hippocampus (Wei et al., 2022; Zhang Y. et al., 2022; Zhang Z. et al., 2022; Zhang et al., 2023a,b). These observations suggest that inflammation and synaptic function are involved in prenatal inflammation- or maternal sleep deprivation-induced anxiety, depression, and cognitive deficits.

In this study, we hypothesized that maternal sleep deprivation aggravates prenatal inflammation-induced anxiety- and depression-like behaviors and cognitive impairment in offspring by modulating inflammatory responses and synaptic function. To test this possibility, pregnant mice were first treated with LPS to induce an inflammatory insult and were then subjected to sleep deprivation to mimic sleep dysfunction. Subsequently, anxiety- and depression-like behaviors and spatial learning and memory function were evaluated in the offspring, as were the hippocampal levels of markers of inflammation (IL-1β, IL-6, TNF-α) and synaptic function (PSD-95, SYN).

## 2. Materials and methods

### 2.1. Animals

**FIGURE 1:**
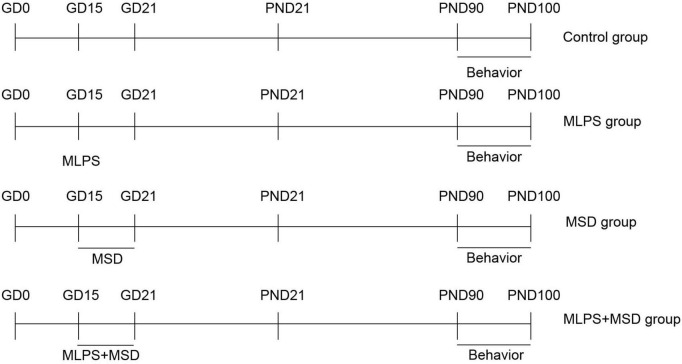
Timeline of the experimental protocol. GD, gestational day; PND, postnatal day; MLPS, maternal intraperitoneal injection of LPS; MSD, maternal sleep deprivation.

c57BL/6J mice (8 weeks old) were purchased from Beijing Vital River Laboratory Animal Device Co., Ltd. Before the experiment, the animals were acclimatized for 7 days under standard laboratory conditions [ambient temperature: 22–25°C, relative humidity: 50 ± 5%, 12-h/12-h light/dark cycle (lights on at 08:00 h)]. Mating was carried out in a cage at a male: female ratio of 1:2 at 21:00 h when it was conducive to mating due to the mice being in active phase and low sleep tension. At the same time, we did not turn on the lights during the dark period, we mated under a red-light source with a brightness of 30 lx. Fertilization was confirmed by the presence of a vaginal plug and was marked as GD0. Pregnant mice were housed individually in cages and were provided with standard rodent feed and purified water *ad libitum*. On GD15, the pregnant mice were intraperitoneally injected with LPS (Abcam LPS, Shanghai Universal Biotech Co., Ltd., Shanghai, China) at a dose of 50 μg/kg or saline and were subjected or not to sleep deprivation for 6 h daily until GD21 (Yan et al., 2022). The day of delivery was designated as postnatal day 0 (PND 0). After weaning, the offspring of the pregnant mice were randomly assigned to the following four groups (*n* = 8 per group), one offspring mouse was randomly selected from each litter: a control group, a maternal intraperitoneal injection of LPS (MLPS) group, a maternal sleep deprivation (MSD) group, and a MLPS + MSD group. The experiments were performed when the offspring reached 3 months of age. All behavioral experiments were conducted from 13:00 to 18:00. All experimental procedures involving animals were approved by the Laboratory Animal Committee of Anhui Medical University (Figure 1).

### 2.2. Sleep deprivation

For sleep deprivation, pregnant mice were placed in a sleep deprivation machine (BW-NSD404, Shanghai Bio-will Co., Ltd.) for 6 h (12:00–18:00 h) during late gestation (GD15–21). Sleep deprivation was ensured by the continuous working of the running belt in the machine (running speed: 0.5 m/min). Mice not subjected to sleep deprivation were also placed in a sleep deprivation machine (BW-NSD404) but with the running belt switched off (0 m/min). Food and water were provided throughout the sleep deprivation period (Wei et al., 2022).

### 2.3. Open field test (OFT)

On PND 90, the locomotor activity and anxiety-like behaviors of the offspring were assessed *via* the OFT. The apparatus consisted of a large black wooden square box (50 cm × 50 cm × 25 cm). The mice were individually placed in the center of the box and allowed to freely explore the environment for 5 min. The experiment was conducted under dim lights (30 lx). The time spent in the central area, the number of entries into the central area, and the total distance moved were recorded and analyzed using ANY-maze software. The box was cleaned with 75% alcohol after each test to eliminate the odor of the previous mouse.

### 2.4. Elevated plus maze (EPM) test

On PND 91, the anxiety-like behaviors of the mice were evaluated using the EPM test. The experimental instrument consisted of a cross-shaped platform with two open arms, two closed arms, and a central area, and was raised 80 cm above the ground. The offspring were placed in the central area of the apparatus with their head facing the open arms and were allowed to freely explore the maze for 6 min. The experiment was conducted under dim lights (30 lx). The time spent in and the number of entries into each arm were recorded and analyzed using the ANY-maze video tracking system. After each recording, the maze was cleaned with 75% alcohol to eliminate the odor of the previous mouse.

### 2.5. Tail suspension test (TST)

On PND 92, behavioral despair in the mice were assessed *via* the TST. The mice were suspended by the tail with adhesive tape, 50 cm above the bench, for 6 min. The immobility time in the last 4 min of the test was recorded. Immobility is a depression-like behavior. Mice were considered to be immobile when they were suspended completely motionless.

### 2.6. Forced swimming test (FST)

On PND 93, behavioral despair in the mice were investigated using the FST. The mice were placed in a glass cylinder (20 cm × 40 cm) filled with 25 ± 1°C water to a depth of 30 cm for 6 min and the immobility time in the last 4 min was quantified. Mice were considered immobile when they were floating passively in an upright position and made only those movements necessary to keep their nose above the water.

### 2.7. Morris water maze (MWM) test

On PND 94, the spatial learning and memory abilities of the offspring were studied using the MWM test. The experiment was carried out for 7 successive days and was performed as previously described (Zhang et al., 2023a). The equipment consisted of a circular black water tank (150 cm in diameter, 30 cm in height), a submerged black escape platform (10 cm in diameter, 24 cm in height), and a camera with a video recording system on the ceiling to analyze the activity of mice in the tank. The tank was divided into four quadrants, each with specific visual cues. The test comprised two parts, namely, a learning phase and a memory phase. In the learning phase, each offspring was separately and randomly placed in a quadrant with their heads facing the wall and was allowed 60 s to find the platform. If a mouse failed to find the platform within the specified time, it was guided to the platform and allowed to stay there for 30 s. Four trials were conducted per day with a 15-min inter-trial interval and the learning phase lasted for 7 days. The escape latency, distance swam, and swimming velocity were recorded and analyzed with the ANY-maze tracking system (Stoelting, USA). In the memory phase, the probe trial was performed 2 h after the final trial of the learning phase. For this trial, the platform was removed, and the mice were placed in the tank in the quadrant opposite the target quadrant and were allowed to freely explore for 60 s. The percentage of time spent and the distance swam in the target quadrant were analyzed using ANY-Maze software (Stoelting, USA).

### 2.8. Tissue preparation

At PND 101, the mice were deeply anesthetized with 2% sodium pentobarbital, and the hippocampus was harvested on ice, snap-frozen in liquid nitrogen, and stored at −80°C for ELISA, western blot, and RT-PCR analysis.

### 2.9. ELISA

Hippocampal tissues of offspring were homogenized in PBS buffer and centrifuged at 1,500 × *g* for 10 min at 4°C. The levels of IL-1β, IL-6, and TNF-α in the resulting supernatants were detected using the respective ELISA kits following the manufacturer’s instructions.

### 2.10. Western blotting

The hippocampus was collected from offspring and lysed in RIPA lysis buffer. After centrifugation at 12,000 rpm for 15 min, the supernatant was harvested, 5 × SDS-PAGE protein loading buffer (1:4) was added to the supernatant, and the proteins were denatured by placing the mixture in a boiling water bath for 15 min. Then, 5–10 μL of the protein sample was added to each of the wells of the SDS-PAGE gel, and electrophoresis was performed at a constant voltage (80 V) for approximately 1 h. The proteins were then transferred to a membrane and, after blocking with 5% non-fat milk for 2 h at room temperature, the membranes were incubated overnight at 4°C with primary antibodies (rabbit anti-PSD-95, 1:2,000; Abcam, Cambridge, UK; rabbit anti-SYN, 1:1,000; Bioss, Beijing, China). The next day, the membranes were washed three times with PBST (10 min each wash), and then incubated for 1.2 h with secondary antibody [horseradish peroxidase (HRP)-labeled goat anti-rabbit IgG; Zsbio, ZB-2301] at 37 °C, and then washed again three times with PBST, 10 min each wash. The protein bands were imaged using ImageJ software (Media Cybernetics, USA).

### 2.11. Real-time fluorescence-based quantitative PCR

Total RNA was extracted from hippocampal tissue using Trizol reagent and reverse transcribed into cDNA using a RT-PCR kit (TaKaRa, RR047A, Japan). The resulting cDNA was used as a template for fluorescence quantification. The qPCR cycling program consisted of one cycle of pre-denaturation for 1 min at 95°C, followed by 40 cycles of 20 s at 95°C and 1 min at 60°C. The relative mRNA levels of the target genes were calculated using the 2^–Δ^ ^Δ^ ^Ct^ method. The sequences of the primers used for PCR are shown in Table 1.

**TABLE 1:** Sequences of the primers used for qPCR.

Gene	Amplicon size (bp)	Forward primer (5′→ 3′)	Reverse primer (5′→ 3′)
β-actin	120	AGTGTGACGTTGACATCCGT	TGCTAGGAGCCAGAGCAGTA
PSD-95	110	GCTCCCTGGAGAATGTGCTA	TGAGAAGCACTCCGTGAACT
SYN	124	GCCTACCTTCTCCACCCTTT	GCACTACCAACGTCACAGAC

### 2.12. Statistical analyses

All data was normal and homogeneous and was presented as means ± standard error of the mean (SEM). Statistical analysis was performed in GraphPad Prism 8.0 and SPSS 23.0. The data for the MWM test outcomes were analyzed using a repeated measures ANOVA with two factors. One-way ANOVA followed by Tukey’s least-significant difference *post-hoc* test was employed for multiple group comparisons. *P*-values < 0.05 were considered significant. Correlations were assessed using Pearson’s correlation coefficient.

## 3. Results

### 3.1. Maternal sleep deprivation increased anxiety-like behavior caused by MLPS in the OFT

**FIGURE 2:**
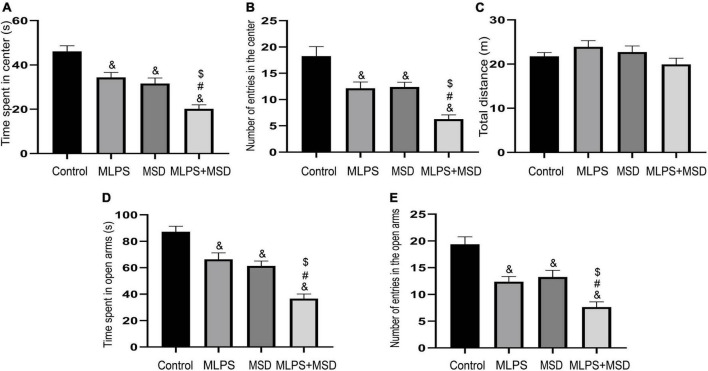
Assessment of anxiety-like behavior using the open field test and the elevated plus maze test. **(A)** The time spent in the central area in the open field test. **(B)** The number of entries into the central area in the open field test. **(C)** The total distance moved in the open field test. **(D)** The time spent in the open arms in the elevated plus maze test. **(E)** The number of entries into the open arms in the elevated plus maze test. Data are presented as means ± SEM (*n* = 8 per group). ^&^*P* < 0.05 vs. the control group; ^#^*P* < 0.05 vs. the MLPS group; ^$^*P* < 0.05 vs. the MSD group.

The one-way ANOVA results showed that the time spent in and the number of entries into the central area differed markedly among the four groups of mice [time spent in the central area: treatment: *F*_(3, 28)_ = 21.06, *P* < 0.01; number of entries in the center: treatment: *F*_(3, 28)_ = 15.12, *P* < 0.01]. *Post hoc* analysis indicated that mice in the MLPS, MSD, and MLPS + MSD groups spent significantly less time in and had substantially fewer entries into the central area in comparison with mice in the control group (*Ps* < 0.05). Furthermore, our data revealed that the time spent in and the number of entries into the central area were both lower among offspring of the MSD + MLPS group than among those of either the MLPS or MSD groups (*Ps* < 0.05). However, no difference in these parameters was observed between the MLPS and MSD groups (*P* > 0.05) (Figures 2A–C). Additionally, no difference in the total distance moved in the OFT was recorded among the four groups [treatment: *F*_(3, 28)_ = 1.76, *P* > 0.05].

### 3.2. Maternal sleep deprivation enhanced MLPS treatment-induced anxiety in the EPM test

The one-way ANOVA showed significant effects of treatment for time spent in and number of entries into the open arms in the EPM test [time spent in the open arms: *F*_(3, 28)_ = 25.81, *P* < 0.01; entries into the open arms: *F*_(3, 28)_ = 17.21, *P* < 0.01]. The results indicated that, compared with offspring in the control group, those in the MLPS, MSD, and MLPS + MSD groups spent markedly less time in and had substantially fewer number of entries into the open arms of the EPM (*Ps* < 0.01). Moreover, *post hoc* analysis showed that the time spent in and the number of entries into the open arms were reduced in the MSD + MLPS group compared with that seen in the MLPS or MSD groups (*Ps* < 0.05). However, no difference in either parameter was detected between the MLPS and MSD groups (*P* > 0.05) (Figures 2D, E).

### 3.3. Maternal sleep deprivation augmented the depression-like behavior caused by MLPS treatment in the FST

**FIGURE 3:**
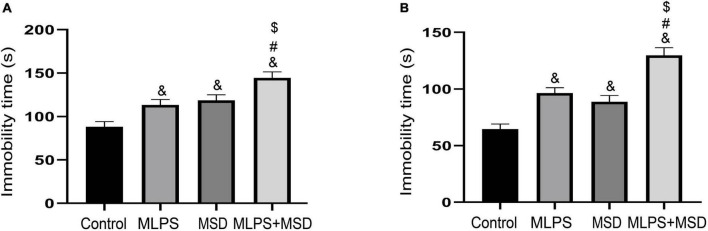
Assessment of depression-like behavior using the tail suspension test and the forced swimming test. **(A)** The immobility time of offspring in the four groups in the forced swimming test. **(B)** The immobility time of offspring in the four groups in the elevated plus maze test. Data are presented as means ± SEM (*n* = 8 per group). ^&^*P* < 0.05 vs. the control group; ^#^*P* < 0.05 vs. the MLPS group; ^$^*P* < 0.05 vs. the MSD group.

In the FST, the one-way ANOVA for immobility time demonstrated significant effects of treatment [*F*_(3, 28)_ = 12.98, *P* < 0.01]. Our data showed that immobility time was significantly increased in the MLPS, MSD, and MLPS + MSD groups compared with that in the control group (*Ps* < 0.05). Additionally, the immobility time was significantly longer in the MLPS + MSD group than in the MLPS and MSD groups (*Ps* < 0.05). However, there was no difference in immobility time between the MLPS and MSD groups (*P* > 0.05) (Figure 3A).

### 3.4. Maternal sleep deprivation heightened the MLPS treatment-induced depression-like behavior in the TST

In the TST, the one-way ANOVA for immobility time showed significant effects of treatment [*F*_(3, 28)_ = 24.07, *P* < 0.01]. *Post hoc* analysis revealed that immobility time differed significantly between the MLPS, MSD, and MLPS + MSD groups when compared with that in the control group (*Ps* < 0.05). Compared with the MLPS or MSD group, immobility time was significantly increased in the MLPS + MSD group (*Ps* < 0.01) (Figure 3B). Collectively, these results suggested maternal sleep deprivation exacerbated the anxiety- and depression-like behaviors induced by prenatal LPS exposure in the offspring mice.

### 3.5. Maternal sleep deprivation aggravated the cognitive dysfunction caused by MLPS treatment in the MWM test

#### 3.5.1. Learning phase

**FIGURE 4:**
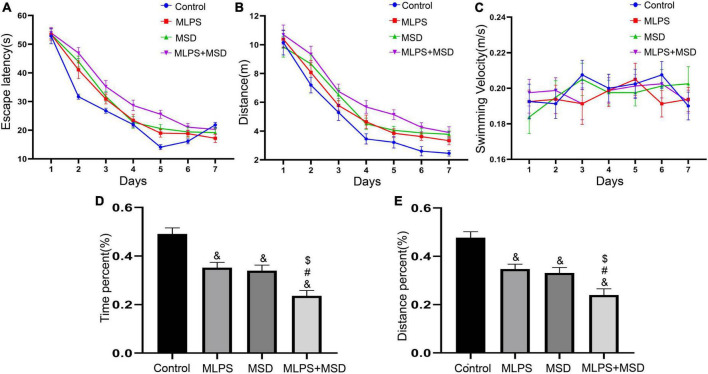
Assessment of cognitive function using the Morris water maze test. **(A)** The escape latency in the learning phase. **(B)** The distance swam in the learning phase. **(C)** The swimming velocity in the learning phase. **(D)** The percent time spent in the target quadrant in the memory phase. **(E)** The percent distance swam in the target quadrant in the memory phase. Data are presented as means ± SEM (*n* = 8 per group). ^&^*P* < 0.05 vs. the control group; ^#^*P* < 0.05 vs. the MLPS group; ^$^*P* < 0.05 vs. the MSD group.

The mixed ANOVA for escape latency and the distance swam showed significant effects of day, treatment, and day × treatment interaction [escape latency: time: *F*_(6, 168)_ = 240.50, *P* < 0.01, treatment: *F*_(3, 28)_ = 17.63, *P* < 0.01, interaction: *F*_(18, 168)_ = 2.49, *P* < 0.05; distance swam: time: *F*_(6, 168)_ = 117.10, *P* < 0.01, treatment: *F*_(3, 28)_ = 16.16, *P* < 0.01, interaction: *F*_(18, 168)_ = 0.45, *P* > 0.05]. Further analysis indicated that the escape latency and distance swam were both increased in the MLPS, MSD, and MLPS + MSD groups in comparison with that seen in the control group (*Ps* < 0.05). Additionally, compared with mice in the MLPS or MSD group, those in the MLPS + MSD group exhibited significantly longer escape latency and swam longer distances (*Ps* < 0.05). However, there was no difference between the MLPS and MSD groups in either parameter (*P* > 0.05) (Figures 4A–C). The repeated measures ANOVA with two factors for swimming velocity demonstrated no significant effects of day, treatment and day × treatment interaction [time: *F*_(6, 168)_ = 0.78, *P* = 0.58, treatment: *F*_(3, 28)_ = 0.23, *P* = 0.87, interaction: *F*_(18, 168)_ = 0.46, *P* > 0.05].

#### 3.5.2. Memory phase

The one-way ANOVA showed that there was a significant effect of treatment on the percentage of time spent and the distance swam in the target quadrant in the MWM test [percent distance: *F*_(3, 28)_ = 18.14, *P* < 0.01; percent time: *F*_(3, 28)_ = 21.73, *P* < 0.01]. The results showed that the percentage of time spent and the distance swam in the target quadrant were significantly reduced in the MLPS, MSD, and MLPS + MSD groups relative to that seen in the control group (*Ps* < 0.01). Moreover, both parameters were significantly lower in the MLPS + MSD group than in the MLPS and MSD groups (*Ps* < 0.05). However, there was no difference between the MLPS and MSD groups regarding either the percent of time spent or the distance swam in the target quadrant (*P* > 0.05) (Figures 4D, E). These results indicated maternal sleep deprivation exacerbated the learning and memory impairment induced by prenatal LPS exposure in the offspring mice.

### 3.6. The effect of maternal LPS treatment and maternal sleep deprivation on the hippocampal levels of IL-1β, IL-6, and TNF-α

**FIGURE 5:**
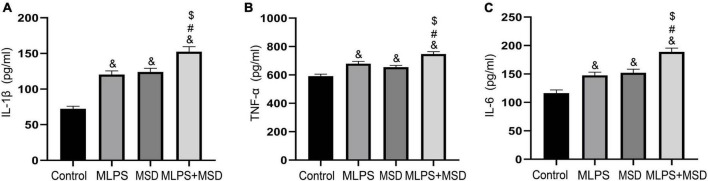
**(A)** The levels of IL-1β in the hippocampus of offspring in the four groups. **(B)** The levels of TNF-α in the hippocampus of offspring in the four groups. **(C)** The levels of IL-6 in the hippocampus of offspring in the four groups. Data are presented as means ± SEM (*n* = 8 per group). ^&^*P* < 0.05 vs. the control group; ^#^*P* < 0.05 vs. the MLPS group; ^$^*P* < 0.05 vs. the MSD group.

We investigated the effect of maternal sleep deprivation and maternal LPS treatment on the levels of the inflammatory factors IL-1β, IL-6, and TNF-α in the hippocampus. The one-way ANOVA showed significant effect of treatment on the levels of all three factors [IL-1β: *F*_(3, 28)_ = 38.82, *P* < 0.01; IL-6: *F*_(3, 28)_ = 24.40, *P* < 0.01; TNF-α: *F*_(3, 28)_ = 19.13, *P* < 0.01]. The hippocampal levels of IL-1β, IL-6, and TNF-α were greatly increased in the MLPS, MSD, and MLPS + MSD groups in comparison with those in the control group (*Ps* < 0.05). Moreover, the levels of these inflammatory factors were relatively higher in the MLPS + MSD group than in the MLPS or MSD group (*Ps* < 0.05). However, no differences in the hippocampal levels of IL-1β, IL-6, and TNF-α were detected between the MLPS and MSD groups (*Ps* > 0.05) (Figures 5A–C). These results indicated that maternal sleep deprivation further increased the elevated proinflammatory cytokines induced by prenatal LPS exposure.

### 3.7. The effect of maternal LPS treatment and maternal sleep deprivation on the expression of PSD-95 and SYN in the hippocampus

**FIGURE 6:**
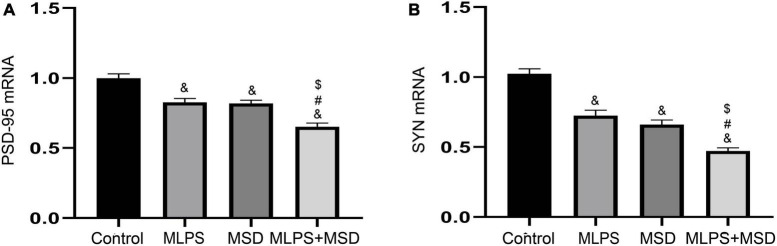
The relative mRNA levels of PSD-95 and SYN in the hippocampus of offspring in the four groups. **(A)** The hippocampal expression levels of PSD-95 mRNA. **(B)** The hippocampal expression levels of SYN mRNA. Data are presented as means ± SEM (*n* = 8 per group). ^&^*P* < 0.05 vs. the control group; ^#^*P* < 0.05 vs. the MLPS group; ^$^*P* < 0.05 vs. the MSD group.

The one-way ANOVA demonstrated that the treatments had a significant effect on the mRNA levels of PSD-95 and SYN in the hippocampus [PSD-95 mRNA: *F*_(3, 28)_ = 26.22, *P* < 0.01; SYN mRNA: *F*_(3, 28)_ = 48.57, *P* < 0.01]. As shown in Figure 6, the relative mRNA levels of PSD-95 and SYN were significantly reduced in the MLPS, MSD, and MLPS + MSD groups compared with those of the control group (*Ps* < 0.01). Furthermore, Tukey’s *post-hoc* tests revealed that the mRNA levels of PSD-95 and SYN in the MLPS + MSD group were significantly lower than those in the MLPS or MSD group (*Ps* < 0.01). No differences in the mRNA levels of PSD-95 and SYN mRNA were found between the MLPS and MSD groups (*Ps* > 0.05) (Figures 6A, B).

**FIGURE 7:**
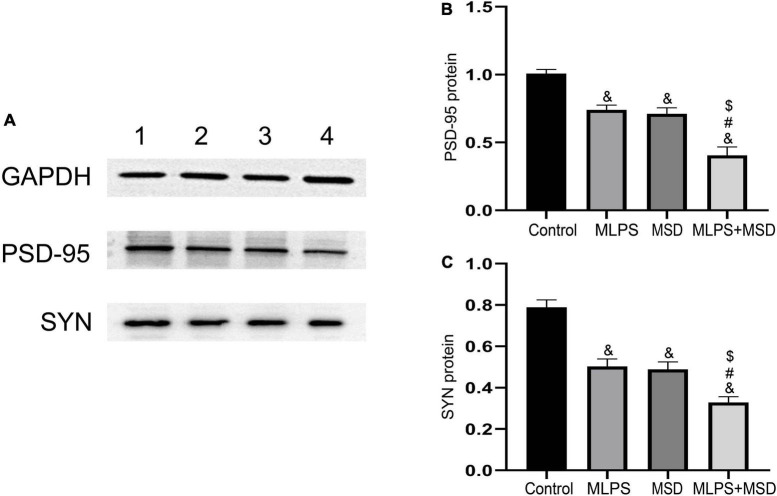
Relative protein levels of PSD-95 and SYN in the hippocampus of offspring in the four groups. **(A)** Western blot analysis of PSD-95 and SYN protein expression in the hippocampus of offspring in the four groups. **(B)** Quantitative analysis of the PSD-95 protein level in the hippocampus. **(C)** Quantitative analysis of the SYN protein level in the hippocampus. Data are presented as means ± SEM (*n* = 6 per group). ^&^*P* < 0.05 vs. the control group; ^#^*P* < 0.05 vs. the MLPS group; ^$^*P* < 0.05 vs. the MSD group.

The one-way ANOVA showed that there was a significant effect of treatments on the protein expression of PSD-95 and SYN in the hippocampus [PSD-95: *F*_(3, 20)_ = 31.61, *P* < 0.01; SYN: *F*_(3, 20)_ = 30.68, *P* < 0.01]. One-way ANOVA indicated that the protein levels of PSD-95 and SYN were significantly lower in mice of the MLPS, MSD, and MLPS + MSD groups than in those of the control group (*Ps* < 0.01). Moreover, Tukey’s *post-hoc* tests revealed that the levels of both proteins were significantly lower in the MLPS + MSD group than in the MLPS or MSD group (*Ps* < 0.05). However, the hippocampal protein levels of PSD-95 and SYN did not differ between the MLPS and MSD groups (*Ps* > 0.05) (Figures 7A–C). These results indicated that maternal sleep deprivation further exacerbated the downregulated synaptic proteins induced by prenatal LPS exposure.

### 3.8. Correlations between anxiety-/depression-/cognitive deficit-like behaviors and the hippocampal expression levels of IL-1β, IL-6, and TNF-α

Pearson’s correlation analysis revealed that the hippocampal levels of IL-1β, IL-6, and TNF-α were negatively correlated with the time spent in and the number of entries into the central area in the MLPS, MSD, and MLPS + MSD groups in both the OFT (*Ps* < 0.05) and the EPM test (*Ps* < 0.05). Additionally, in the MWM test, the hippocampal levels of the three proinflammatory factors were positively correlated with escape latency and the distance swam in the learning phase and negatively correlated with the percentage of time spent and distance swam in the target quadrant in the memory phase in the MLPS, MSD, and MLPS + MSD groups (*Ps* < 0.05) (Table 2).

**TABLE 2:** Pearson correlations between anxiety-/depression-/cognition deficit-like behaviors and the levels of inflammatory factors (IL-1β, TNF-α, and IL-6) in the hippocampus [r (p)].

Task	Index	Group	Inflammatory factor		
			IL-1β	TNF-α	IL-6
Open field test	Time spent in the central area	Control	−0.826 (0.012)*	−0.694 (0.056)	−0.590 (0.123)
		MLPS	−0.873 (0.005)**	−0.811 (0.015)*	−0.767 (0.026)*
		MSD	−0.882 (0.004)**	−0.919 (0.001)**	−0.910 (0.002)**
		MLPS + MSD	−0.850 (0.007)**	−0.799 (0.017)*	−0.819 (0.013)*
	Number of entries into the central area	Control	−0.654 (0.079)	−0.506 (0.201)	−0.304 (0.464)
		MLPS	−0.853 (0.007)**	−0.795 (0.018)*	−0.799 (0.017)*
		MSD	−0.792 (0.019)*	−0.896 (0.003)**	−0.834 (0.010)*
		MLPS + MSD	−0.776 (0.024)*	−0.891 (0.003)**	−0.811 (0.015)*
Elevated plus maze test	Time spent in the open arms	Control	−0.841 (0.009)**	−0.738 (0.036)*	−0.616 (0.104)
		MLPS	−0.848 (0.008)**	−0.822 (0.012)*	−0.762 (0.028)*
		MSD	−0.756 (0.030)*	−0.821 (0.012)*	−0.782 (0.022)*
		MLPS + MSD	−0.853 (0.007)**	−0.826 (0.011)*	−0.932 (0.001)**
	Number of entries into the open arms	Control	−0.515 (0.191)	−0.402 (0.311)	−0.186 (0.660)
		MLPS	−0.862 (0.006)**	−0.833 (0.010)*	−0.841 (0.009)**
		MSD	−0.783 (0.021)*	−0.837 (0.010)**	−0.793 (0.019)*
		MLPS + MSD	−0.821 (0.013)*	−0.813 (0.014)*	−0.862 (0.006)**
Forced swimming test	Immobility time	Control	0.786 (0.021)*	0.608 (0.110)	0.490 (0.218)
		MLPS	0.895 (0.003)**	0.816 (0.013)*	0.835 (0.010)**
		MSD	0.766 (0.027)*	0.819 (0.013)*	0.782 (0.022)*
		MLPS + MSD	0.817 (0.013)*	0.903 (0.002)**	0.773 (0.024)*
Tail suspension test	Immobility time	Control	0.682 (0.063)	0.376 (0.359)	0.279 (0.503)
		MLPS	0.926 (0.001)**	0.837 (0.010)**	0.857 (0.007)**
		MSD	0.799 (0.017)*	0.933 (0.001)**	0.837 (0.010)**
		MLPS + MSD	0.896 (0.003)**	0.851 (0.007)**	0.842 (0.009)**
Morris water maze test	Escape latency	Control	0.804 (0.016)*	0.608 (0.110)	0.595 (0.120)
		MLPS	0.820 (0.013)*	0.731 (0.039)*	0.733 (0.039)*
		MSD	0.764 (0.027)*	0.812 (0.014)*	0.778 (0.023)*
		MLPS + MSD	0.855 (0.007)**	0.819 (0.013)*	0.818 (0.013)*
	Distance swam	Control	0.828 (0.011)*	0.779 (0.023)*	0.572 (0.138)
		MLPS	0.911 (0.002)**	0.868 (0.005)**	0.816 (0.013)*
		MSD	0.824 (0.012)*	0.879 (0.004)**	0.848 (0.008)**
		MLPS + MSD	0.891 (0.003)**	0.775 (0.024)*	0.749 (0.032)*
	Percentage of time swam	Control	−0.750 (0.032)*	−0.700 (0.053)	−0.435 (0.281)
		MLPS	−0.898 (0.002)**	−0.900 (0.002)**	−0.824 (0.012)*
		MSD	−0.731 (0.039)*	−0.800 (0.017)*	−0.739 (0.036)*
		MLPS + MSD	−0.766 (0.027)*	−0.847 (0.008)**	−0.744 (0.034)*
	Percentage of distance swam	Control	−0.833 (0.010)*	−0.492 (0.215)	−0.506 (0.200)
		MLPS	−0.904 (0.002)**	−0.892 (0.003)**	−0.827 (0.011)*
		MSD	−0.733 (0.039)*	−0.808 (0.015)*	−0.745 (0.034)*
		MLPS + MSD	−0.791 (0.019)*	−0.798 (0.018)*	−0.766 (0.027)*

*P < 0.05, **P < 0.01. MLPS, maternal intraperitoneal injection of LPS; MSD, maternal sleep deprivation.

### 3.9. Correlations between anxiety-/depression-/cognitive deficit-like behaviors and hippocampal expression levels of PSD-95 and SYN

#### 3.9.1. Correlations with PSD-95 and SYN protein levels

In the OFT, the protein levels of PSD-95 and SYN in the hippocampus were found to be positively correlated with the time spent in and the number of entries into the central area during the OFT in the MLPS, MSD, and MLPS + MSD groups (*Ps* < 0.05). Meanwhile, in the EPM test, the hippocampal protein levels of PSD-95 and SYN were also positively correlated with the time spent in and the number of entries into the open arms in the MLPS, MSD, and MLPS + MSD groups (*Ps* < 0.05). Furthermore, in the learning phase of the MWM test, the hippocampal protein levels of PSD-95 and SYN were negatively correlated with the escape latency and the distance swam (*Ps* < 0.05); in the memory phase of the MWM test, meanwhile, the hippocampal levels of both proteins were positively correlated with the percentage of time spent and distance swam in the target quadrant in the MLPS, MSD, and MLPS + MSD groups (*Ps* < 0.05) (Table 3).

**TABLE 3:** Pearson correlations between anxiety-/depression-/cognition-like behaviors and hippocampal protein levels of PSD-95 and SYN [r (p)].

Task	Index	Group	Synaptic protein	
			PSD-95	SYN
Open field test	Time spent in the central area	Control	0.851 (0.032)*	0.876 (0.022)*
		MLPS	0.936 (0.006)**	0.961 (0.002)**
		MSD	0.938 (0.006)**	0.926 (0.008)**
		MLPS + MSD	0.937 (0.006)**	0.933 (0.007)**
	Number of entries into the central area	Control	0.730 (0.100)	0.737 (0.094)
		MLPS	0.986 (0.000)**	0.893 (0.017)*
		MSD	0.949 (0.004)**	0.835 (0.039)*
		MLPS + MSD	0.962 (0.002)**	0.840 (0.036)*
Elevated plus maze test	Time spent in the open arms	Control	0.760 (0.079)	0.902 (0.014)*
		MLPS	0.855 (0.030)*	0.866 (0.026)*
		MSD	0.896 (0.016)*	0.906 (0.013)*
		MLPS + MSD	0.942 (0.005)**	0.853 (0.031)*
	Number of entries into the open arms	Control	0.571 (0.236)	0.585 (0.222)
		MLPS	0.955 (0.003)**	0.857 (0.029)*
		MSD	0.878 (0.021)*	0.950 (0.004)**
		MLPS + MSD	0.955 (0.003)**	0.897 (0.015)*
Forced swimming test	Immobility time	Control	−0.902 (0.014)*	−0.822 (0.045)*
		MLPS	−0.919 (0.010)**	−0.909 (0.012)*
		MSD	−0.875 (0.022)*	−0.969 (0.001)**
		MLPS + MSD	−0.881 (0.020)*	−0.915 (0.011)*
Tail suspension test	Immobility time	Control	−0.868 (0.025)*	−0.697 (0.124)
		MLPS	−0.968 (0.001)**	−0.940 (0.005)**
		MSD	−0.965 (0.002)**	−0.902 (0.014)*
		MLPS + MSD	−0.857 (0.029)*	−0.936 (0.006)**
Morris water maze test	Escape latency	Control	−0.947 (0.004)**	−0.869 (0.025)*
		MLPS	−0.947 (0.004)**	−0.896 (0.016)*
		MSD	−0.912 (0.011)*	−0.940 (0.005)**
		MLPS + MSD	−0.942 (0.005)**	−0.848 (0.033)*
	Distance swam	Control	−0.792 (0.061)	−0.930 (0.007)**
		MLPS	−0.809 (0.051)	−0.943 (0.005)**
		MSD	−0.898 (0.015)*	−0.963 (0.002)**
		MLPS + MSD	−0.837 (0.038)*	−0.864 (0.027)*
	Percentage of time swam	Control	0.823 (0.044)*	0.853 (0.031)*
		MLPS	0.948 (0.004)**	0.947 (0.004)**
		MSD	0.870 (0.024)*	0.931 (0.007)**
		MLPS + MSD	0.947 (0.004)**	0.891 (0.017)*
	Percentage of distance swam	Control	0.940 (0.005)**	0.816 (0.048)*
		MLPS	0.935 (0.006)**	0.954 (0.003)**
		MSD	0.870 (0.024)*	0.931 (0.007)**
		MLPS + MSD	0.977 (0.001)**	0.869 (0.025)*

*P < 0.05, **P < 0.01. MLPS, maternal intraperitoneal injection of LPS; MSD, maternal sleep deprivation.

#### 3.9.2. Correlations with the mRNA levels of PSD-95 and SYN

Linear correlation analysis revealed that the hippocampal mRNA levels of PSD-95 and SYN were positively correlated with the time spent in and the number of entries into the central area in the MLPS, MSD, MLPS + MSD groups (*Ps* < 0.05) in the OFT. Meanwhile, in the EPM test, the hippocampal mRNA levels of PSD-95 and SYN showed a positive correlation with the time spent in and the number of entries into the central area in the MLPS, MSD, and MLPS + MSD groups (*Ps* < 0.05). Additionally, in the MWM test, the mRNA levels of PSD-95 and SYN in the hippocampus exhibited a negative correlation with the escape latency and the distance swam in the learning phase and a positive correlation with the percentage of time spent and distance swam in the target quadrant in the memory phase in the MLPS, MSD, and MLPS + MSD groups (*Ps* < 0.05) (Table 4).

**TABLE 4:** Pearson correlations between anxiety-/depression-/cognition-like behaviors and hippocampal mRNA expression levels of PSD-95 and SYN [r (p)].

Task	Index	Group	mRNA	
			PSD-95	SYN
Open field test	Time spent in the central area	Control	0.727 (0.041)*	0.740 (0.036)*
		MLPS	0.799 (0.017)*	0.827 (0.011)*
		MSD	0.895 (0.003)**	0.909 (0.002)**
		MLPS + MSD	0.825 (0.012)*	0.844 (0.008)**
	Number of entries into the central area	Control	0.565 (0.144)	0.527 (0.180)
		MLPS	0.834 (0.010)*	0.871 (0.005)**
		MSD	0.779 (0.023)*	0.925 (0.001)**
		MLPS + MSD	0.808 (0.015)*	0.882 (0.004)**
Elevated plus maze test	Time spent in the open arms	Control	0.729 (0.040)*	0.737 (0.037)*
		MLPS	0.786 (0.021)*	0.735 (0.038)*
		MSD	0.785 (0.021)*	0.887 (0.003)**
		MLPS + MSD	0.927 (0.001)**	0.898 (0.002)**
	Number of entries into the open arms	Control	0.406 (0.318)	0.337 (0.414)
		MLPS	0.878 (0.004)**	0.833 (0.010)*
		MSD	0.825 (0.012)*	0.893 (0.003)**
		MLPS + MSD	0.890 (0.003)**	0.885 (0.004)**
Forced swimming test	Immobility time	Control	−0.711 (0.048)*	−0.672 (0.068)
		MLPS	−0.875 (0.004)**	−0.792 (0.019)*
		MSD	−0.821 (0.012)*	−0.827 (0.011)*
		MLPS + MSD	−0.780 (0.022)*	−0.899 (0.002)**
Tail suspension test	Immobility time	Control	−0.518 (0.189)	−0.429 (0.289)
		MLPS	−0.878 (0.004)**	−0.864 (0.006)**
		MSD	−0.781 (0.022)*	−0.970 (0.000)**
		MLPS + MSD	−0.816 (0.014)*	−0.869 (0.005)**
Morris water maze test	Escape latency	Control	−0.655 (0.078)	−0.730 (0.040)*
		MLPS	−0.791 (0.020)*	−0.793 (0.019)*
		MSD	−0.787 (0.020)*	−0.860 (0.006)**
		MLPS + MSD	−0.818 (0.013)*	−793 (0.019)*
	Distance swam	Control	−0.780 (0.023)*	−0.765 (0.027)*
		MLPS	−0.846 (0.008)**	−0.692 (0.057)
		MSD	−0.868 (0.005)**	−0.896 (0.003)**
		MLPS + MSD	−0.724 (0.042)*	−0.739 (0.036)*
	Percentage of time swam	Control	0.815 (0.014)*	0.717 (0.045)*
		MLPS	0.829 (0.011)*	0.862 (0.006)**
		MSD	0.767 (0.026)*	0.881 (0.004)**
		MLPS + MSD	0.773 (0.024)*	0.856 (0.007)**
	Percentage of distance swam	Control	0.617 (0.103)	0.629 (0.095)
		MLPS	0.846 (0.008)**	0.838 (0.009)**
		MSD	0.770 (0.025)*	0.890 (0.003)**
		MLPS + MSD	0.803 (0.016)	0.825 (0.012)*

*P < 0.05, **P < 0.01. MLPS, maternal intraperitoneal injection of LPS; MSD, maternal sleep deprivation.

## 4. Discussion

Environmental factors that promote pregnancy-associated stress events, such as exposure to inflammation and sleep dysfunction, can potentially affect the structure of the brain and the development of the nervous system in offspring. However, how maternal sleep deprivation in combination with prenatal exposure to inflammation affects emotional and cognitive function in offspring remains unclear. Our results showed that maternal sleep deprivation exacerbated anxiety- and depression-like behaviors and learning and memory impairment associated with prenatal exposure to inflammation. Furthermore, maternal sleep deprivation further exacerbated the upregulated of proinflammatory cytokines and the downregulated of synaptic proteins, respectively, induced by the intraperitoneal injection of LPS during pregnancy. These findings suggested that maternal sleep deprivation significantly aggravated behavioral difficulties induced by prenatal exposure to inflammation in offspring accompanied by altered levels of markers of inflammation and synaptic plasticity.

### 4.1. Maternal sleep deprivation aggravated the neurobehavioral abnormalities induced by prenatal exposure to inflammation

Studies have suggested that stress in early life promotes oxidative stress and inflammation and negatively influences the hypothalamic-pituitary-adrenal axis and the neuroendocrine system in offspring, and these effects have long-lasting consequences for emotional and cognitive behaviors (Krugers and Joëls, 2014; Ciafrè et al., 2020; Cattane et al., 2022). LPS administration during pregnancy significantly increased the levels of anxiety- and depression-like behaviors in offspring in both the EPM test and the FST (Enayati et al., 2012). These behaviors have also been observed in the offspring of mother rats subjected to sleep deprivation during the third trimester-equivalent of pregnancy in humans by gentle manual handing (Radhakrishnan et al., 2015). In full agreement with previous findings, our results showed that maternal sleep deprivation or prenatal exposure to inflammation increased the levels of anxiety and depression in offspring, as evidenced by the results of the OFT, TST, FST, and EPM test. Additionally, both of these stressors have been reported to impair cognitive function in both clinical and preclinical studies. Consistent with these observations, we found that maternal sleep deprivation or exposure to inflammation prenatally prolonged the escape latency and increased the distance swam in the learning phase while decreasing the percentage of time spent and distance swam in the target quadrant in the memory phase of the MWM test. This suggested that both environmental stressors impaired learning and memory function in the offspring of affected mothers.

The binding of LPS to Toll-like receptor 4 (TLR4) triggers an inflammatory response (Ren et al., 2023; Tongaonkar et al., 2023). One study showed that exposure to LPS activated the TLR4 signaling pathway and microglia in pregnant mice and led to autism-like behavior in the offspring (Xiao et al., 2021). These findings indicated that LPS-mediated maternal immune activation may be involved in the occurrence of behavioral abnormalities in offspring. Furthermore, sleep deprivation has also been reported to enhance the inflammatory response following exposure to LPS in adult mice (Xu et al., 2020). In the present study, the results showed that maternal sleep deprivation, as a prenatal stress, aggravated the anxiety- and depression-like symptoms and cognitive deficits induced by prenatal exposure to inflammation in the offspring. Previous study showed that chronic sleep deprivation activates the hypothalamic-pituitary-adrenal axis (HPA) and upregulates corticosterone, which enhances vulnerability to LPS challenge (Ito et al., 2020). We suspect that this may have been due to maternal sleep deprivation further exacerbating LPS-activated maternal immunity via modulating the reaction of HPA.

### 4.2. Maternal sleep deprivation exacerbated the prenatal inflammation-induced proinflammatory response

The hippocampus is an important brain region involved in cognitive function, and its function is vulnerable to various stresses (Grigoryan and Segal, 2016). Elevated levels of proinflammatory cytokines in the hippocampus can negatively affect cell proliferation, differentiation, survival, and synaptic function and lead to anxiety, depression, and cognitive dysfunction (Adebayo et al., 2022; Li et al., 2023). Several studies have shown that the intraperitoneal injection of LPS upregulates the expression of proinflammatory cytokines, including IL-1β, IL-6, and TNF-α, leading to anxiety, depression, and cognitive deficits (Huang et al., 2020; Chen et al., 2021). IL-1β- or TNF-α-knockout mice show reduced levels of anxiety, depression, and cognitive decline relative to that seen in their wild-type counterparts (Goshen et al., 2008; Kaster et al., 2012; Murray et al., 2013; Naude et al., 2014). At the high levels detected following prenatal exposure to inflammation, proinflammatory cytokines can cross the placental and blood-brain barriers and induce an inflammatory response in offspring mice, which is associated with abnormal neurobehaviors. Long-term exercise was demonstrated to improve prenatal exposure to inflammation-induced anxiety and depression in offspring by increasing the concentrations of anti-inflammatory cytokines (Rahimi et al., 2020). Meanwhile, maternal sleep deprivation induced a notable inflammatory response and exerted a significant inhibitory effect on neurogenesis, leading to spatial learning and memory impairment in offspring (Zhao et al., 2014). In the current study, we also found that inflammation contributed to anxiety- and depression-like behaviors and cognitive impairment in offspring following maternal sleep deprivation or prenatal exposure to inflammation, as reflected by the high levels of proinflammatory cytokines detected in the MLPS and MSD groups. Importantly, the expression levels of proinflammatory cytokines were higher in the offspring of mice in the MSD + MLPS group than in those of mice in the MSD or MLPS group, suggesting that maternal sleep deprivation may aggravate the inflammatory response induced by prenatal exposure to inflammation, thereby further impairing emotional and cognitive functioning in the offspring.

### 4.3. Maternal sleep deprivation exacerbated the decrease in the levels of synaptic proteins resulting from prenatal exposure to inflammation

Substantial evidence supports the existence of a direct link between synaptic plasticity and anxiety, depression, and cognitive function (Wang et al., 2021; Hu et al., 2023). Early life is a critical period for synaptic pruning, synaptic maturation, and neural network establishment, and is susceptible to interference from external environmental factors (Malave et al., 2022; Roszkowska et al., 2022). One study reported that exposure to inflammation *in utero* increased the levels of synaptic pruning-associated proteins (C3 and CR3A) and decreased dendrite length and dendritic spine density in offspring mice (Xiao et al., 2021). Maternal sleep deprivation, an early-life stress, was reported to impair long-term potentiation and synaptic transmission in the hippocampi of offspring (Peng et al., 2016). The synaptic proteins PSD-95 and SYN play an important role in dendrite development and participate in synaptic plasticity (Martínez-Torres et al., 2021; Li et al., 2022). In the current study, we found that the mRNA and protein levels of PSD-95 and SYN were decreased in the offspring of mice in the MSD and MLPS groups, indicating that a potential impairment in synapse formation and/or plasticity contributed to the abnormal behaviors in the offspring. Furthermore, the combination of prenatal exposure to inflammation and maternal sleep deprivation aggravated the synaptic dysfunction induced by either stressor alone, as evidenced by the downregulated levels of PSD-95 and SYN in the MLPS + MSD group relative to those in the MLPS and MSD groups. This partly explains the mechanisms involved in how maternal sleep deprivation exacerbates the emotional and cognitive dysfunction induced by exposure to inflammation *in utero*.

### 4.4. Correlation between anxiety-/depression-/cognition-related behaviors and the markers of inflammation and synaptic plasticity

Growing evidence indicates that the levels of proinflammatory cytokines and synaptic proteins are closely related to anxiety, depression, and cognitive decline. Elevated levels of proinflammatory cytokines in the blood are associated with high anxiety and depression scores in patients (Zou et al., 2018; Leff Gelman et al., 2019). In our previous study, we showed that the levels of synaptic proteins are strongly correlated with cognitive dysfunction induced by maternal sleep deprivation (Wei et al., 2022). In the present study, the levels of proinflammatory cytokines and synaptic proteins were correlated with indicators of the OFT, EPM test, FST, TST, and MWM test. This indicated that the anxiety, depression, and cognitive impairment induced by prenatal exposure to inflammation coupled with maternal sleep deprivation may be a consequence, at least in part, of the upregulation of the levels of proinflammatory cytokines and the downregulation of the levels of synaptic proteins in the hippocampus.

Our study had some limitations. First, we only evaluated behavioral phenotypes in male offspring mice. Sex differences relating to the effect of maternal sleep deprivation and prenatal exposure to inflammation on the behavior of offspring were not evaluated. Secondly, this study only documented the association between inflammation and synaptic dysfunction and abnormal behaviors in the offspring. The underlying mechanisms were not explored. Thirdly, we examined the alterations in synaptic proteins and did not evaluate the hippocampal synaptic plasticity including long-term potentiation and long-term depression in the offspring mice. Lastly, the levels of markers of inflammation and synaptic function were determined only in the hippocampus and not in other brain regions associated with anxiety, depression, and cognitive impairment (Moore et al., 2023; Wood et al., 2023).

## 5. Conclusion

In conclusion, this is the first study showing that maternal sleep deprivation significantly aggravates anxiety, depression, and learning and memory impairment induced by intrauterine exposure to inflammation and that this effect is associated with alterations in the levels of markers of inflammation and synaptic function. Our findings suggest that it is important to evaluate sleep status in pregnant women with infection and treat the infection wherever possible.

## Data availability statement

The original contributions presented in this study are included in the article/supplementary material, further inquiries can be directed to the corresponding authors.

## Ethics statement

The animal study was reviewed and approved by the Association of Laboratory Animal Sciences and the Center for Laboratory Animal Sciences of the Anhui Medical University (No. LLSC20190710).

## Author contributions

Y-MZ, M-YZ, and R-MW designed the study, performed the behavioral tests, and drafted the manuscript. J-YZ and K-XZ were responsible for the western blotting, RT-qPCR, and ELISA. B-LL, Y-JG, and X-YK analyzed the data and constructed the graphs. X-YL and G-HC revised the manuscript and are responsible for the data. All authors read and approved the final version of the manuscript.

## References

[B1] Adebayo O. Ben-Azu B. Ajayi A. Wopara I. Aduema W. Kolawole T. (2022). Gingko biloba abrogate lead-induced neurodegeneration in mice hippocampus: Involvement of NF-κB expression, myeloperoxidase activity and pro-inflammatory mediators. *Biol. Trace Elem. Res.* 200 1736–1749. 10.1007/s12011-021-02790-3 34240327

[B2] Al-Abri M. Al-Yaarubi S. Said E. (2023). Circadian rhythm, sleep, and immune response and the fight against COVID-19. *Oman Med. J.* 38:e477. 10.5001/omj.2023.38 37009205 PMC10031746

[B3] Cattane N. Vernon A. Borsini A. Scassellati C. Endres D. Capuron L. (2022). Preclinical animal models of mental illnesses to translate findings from the bench to the bedside: Molecular brain mechanisms and peripheral biomarkers associated to early life stress or immune challenges. *Eur. Neuropsychopharmacol.* 58 55–79. 10.1016/j.euroneuro.2022.02.002 35235897

[B4] Chen L. Qing W. Yi Z. Lin G. Peng Q. Zhou F. (2021). NU9056, a KAT 5 inhibitor, treatment alleviates brain dysfunction by inhibiting NLRP3 inflammasome activation, affecting gut microbiota, and derived metabolites in LPS-treated mice. *Front. Nutr.* 8:701760. 10.3389/fnut.2021.701760 34327209 PMC8313765

[B5] Ciafrè S. Ferraguti G. Greco A. Polimeni A. Ralli M. Ceci F. M. (2020). Alcohol as an early life stressor: Epigenetics, metabolic, neuroendocrine and neurobehavioral implications. *Neurosci. Biobehav. Rev.* 118 654–668. 10.1016/j.neubiorev.2020.08.018 32976915

[B6] Deng D. Cui Y. Gan S. Xie Z. Cui S. Cao K. (2022). Sinisan alleviates depression-like behaviors by regulating mitochondrial function and synaptic plasticity in maternal separation rats. *Phytomedicine* 106:154395. 10.1016/j.phymed.2022.154395 36103769

[B7] Enayati M. Solati J. Hosseini M. Shahi H. Saki G. Salari A. (2012). Maternal infection during late pregnancy increases anxiety- and depression-like behaviors with increasing age in male offspring. *Brain Res. Bull.* 87 295–302. 10.1016/j.brainresbull.2011.08.015 21893170

[B8] Everson C. (1993). Sustained sleep deprivation impairs host defense. *Am. J. Physiol. Regul. Integr. Comp. Physiol.* 265 R1148–R1154. 10.1152/ajpregu.1993.265.5.R1148 8238617

[B9] Everson C. Toth L. (2000). Systemic bacterial invasion induced by sleep deprivation. *Am. J. Physiol. Regul. Integr. Comp. Physiol.* 278 R905–R916. 10.1152/ajpregu.2000.278.4.R905 10749778

[B10] Goshen I. Kreisel T. Ben-Menachem-Zidon O. Licht T. Weidenfeld J. Ben-Hur T. (2008). Brain interleukin-1 mediates chronic stress-induced depression in mice via adrenocortical activation and hippocampal neurogenesis suppression. *Mol. Psychiatry* 13 717–728. 10.1038/sj.mp.4002055 17700577

[B11] Grigoryan G. Segal M. (2016). Lasting differential effects on plasticity induced by prenatal stress in dorsal and ventral hippocampus. *Neural Plast.* 2016:2540462. 10.1155/2016/2540462 26881096 PMC4736977

[B12] Han V. Patel S. Jones H. Dale R. (2021). Maternal immune activation and neuroinflammation in human neurodevelopmental disorders. *Nat. Rev. Neurol.* 17 564–579. 10.1038/s41582-021-00530-8 34341569

[B13] Hao L. Hao X. Li S. Li X. (2010). Prenatal exposure to lipopolysaccharide results in cognitive deficits in age-increasing offspring rats. *Neuroscience* 166 763–770. 10.1016/j.neuroscience.2010.01.006 20074621

[B14] Hu L. Gong Q. Zhou Y. Wang Y. Qiu T. Fang Y. (2023). Arc-mediated synaptic plasticity regulates cognitive function in a migraine mouse model. *Brain Sci.* 13:331. 10.3390/brainsci13020331 36831874 PMC9954307

[B15] Huang J. Shen C. Ye R. Shi Y. Li W. (2021). The effect of early maternal separation combined with adolescent chronic unpredictable mild stress on behavior and synaptic plasticity in adult female rats. *Front. Psychiatry* 12:539299. 10.3389/fpsyt.2021.539299 33746787 PMC7973020

[B16] Huang W. Liu K. Lin S. Chen T. Tseng C. Chen H. (2020). NADPH oxidase 2 as a potential therapeutic target for protection against cognitive deficits following systemic inflammation in mice. *Brain Behav. Immun.* 84 242–252. 10.1016/j.bbi.2019.12.006 31841660

[B17] Irwin M. (2002). Effects of sleep and sleep loss on immunity and cytokines. *Brain Behav. Immun.* 16 503–512. 10.1016/s0889-1591(02)00003-x 12401464

[B18] Ito N. Takemoto H. Hasegawa A. Sugiyama C. Honma K. Nagai T. (2020). Neuroinflammaging underlies emotional disturbances and circadian rhythm disruption in young male senescence-accelerated mouse prone 8 mice. *Exp. Gerontol.* 142:111109. 10.1016/j.exger.2020.111109 33069781

[B19] Izci-Balserak B. Keenan B. Corbitt C. Staley B. Perlis M. Pien G. (2018). Changes in sleep characteristics and breathing parameters during sleep in early and late pregnancy. *J. Clin. Sleep Med.* 14 1161–1168. 10.5664/jcsm.7216 29991418 PMC6040782

[B20] Jia C. Zhang R. Wei L. Xie J. Zhou S. Yin W. (2023). Investigation of the mechanism of tanshinone IIA to improve cognitive function via synaptic plasticity in epileptic rats. *Pharm. Biol.* 61 100–110. 10.1080/13880209.2022.2157843 36548216 PMC9788714

[B21] Kaster M. Gadotti V. Calixto J. Santos A. Rodrigues A. (2012). Depressive-like behavior induced by tumor necrosis factor-α in mice. *Neuropharmacology* 62 419–426. 10.1016/j.neuropharm.2011.08.018 21867719

[B22] Knorr D. Fox M. (2023). An evolutionary perspective on the association between grandmother-mother relationships and maternal mental health among a cohort of pregnant Latina women. *Evol. Hum. Behav.* 44 30–38. 10.1016/j.evolhumbehav.2022.10.005 37065817 PMC10100916

[B23] Krugers H. Joëls M. (2014). Long-lasting consequences of early life stress on brain structure, emotion and cognition. *Curr. Top. Behav. Neurosci.* 18 81–92. 10.1007/7854_2014_289 24862989

[B24] Leff Gelman P. Mancilla-Herrera I. Flores-Ramos M. Saravia Takashima M. Cruz Coronel F. Cruz Fuentes C. (2019). The cytokine profile of women with severe anxiety and depression during pregnancy. *BMC Psychiatry* 19:104. 10.1186/s12888-019-2087-6 30943938 PMC6446269

[B25] Li H. Xiang D. Gong C. Wang X. Liu L. (2023). Naturally derived injectable hydrogels with ROS-scavenging property to protect transplanted stem cell bioactivity for osteoarthritic cartilage repair. *Front. Bioeng. Biotechnol.* 10:1109074. 10.3389/fbioe.2022.1109074 36686241 PMC9848398

[B26] Li Q. Wang X. Wang Z. Lin Z. Yang J. Chen J. (2022). Changes in dendritic complexity and spine morphology following BCG immunization in APP/PS1 mice. *Hum. Vaccin. Immunother.* 18:2121568. 10.1080/21645515.2022.2121568 36113067 PMC9746542

[B27] Malave L. van Dijk M. Anacker C. (2022). Early life adversity shapes neural circuit function during sensitive postnatal developmental periods. *Transl. Psychiatry* 12:306. 10.1038/s41398-022-02092-9 35915071 PMC9343623

[B28] Mansfield R. Cecula P. Pedraz C. Zimianiti I. Elsaddig M. Zhao R. (2023). Impact of perinatal factors on biomarkers of cardiovascular disease risk in preadolescent children. *J. Hypertens.* 41 1059–1067. 10.1097/HJH.0000000000003452 37115847

[B29] Martínez-Torres N. Vázquez-Hernández N. Martín-Amaya-Barajas F. Flores-Soto M. González-Burgos I. (2021). Ibotenic acid induced lesions impair the modulation of dendritic spine plasticity in the prefrontal cortex and amygdala, a phenomenon that underlies working memory and social behavior. *Eur. J. Pharmacol.* 896:173883. 10.1016/j.ejphar.2021.173883 33513334

[B30] Moore T. Medalla M. Ibañez S. Wimmer K. Mojica C. Killiany R. (2023). Neuronal properties of pyramidal cells in lateral prefrontal cortex of the aging rhesus monkey brain are associated with performance deficits on spatial working memory but not executive function. *GeroScience* [Epub ahead of print]. 10.1007/s11357-023-00798-2 37106282 PMC10400510

[B31] Murray C. Obiang P. Bannerman D. Cunningham C. (2013). Endogenous IL-1 in cognitive function and anxiety: A study in IL-1RI-/- mice. *PLoS One* 8:e78385. 10.1371/journal.pone.0078385 24205219 PMC3813582

[B32] Naude P. Dobo N. van der Meer D. Mulder C. Pawironadi K. den Boer J. (2014). Analysis of cognition, motor performance and anxiety in young and aged tumor necrosis factor alpha receptor 1 and 2 deficient mice. *Behav. Brain Res.* 258 43–51. 10.1016/j.bbr.2013.10.006 24135018

[B33] Paris J. Brunton P. Russell J. Frye C. (2011). Immune stress in late pregnant rats decreases length of gestation and fecundity, and alters later cognitive and affective behaviour of surviving pre-adolescent offspring. *Stress* 14 652–664. 10.3109/10253890.2011.628719 21995525 PMC3376536

[B34] Peng Y. Wang W. Tan T. He W. Dong Z. Wang Y. (2016). Maternal sleep deprivation at different stages of pregnancy impairs the emotional and cognitive functions, and suppresses hippocampal long-term potentiation in the offspring rats. *Mol. Brain* 9:17. 10.1186/s13041-016-0197-3 26876533 PMC4753670

[B35] Pluciński M. M. Ngonghala C. N. Getz W. M. Bonds M. H. (2012). Clusters of poverty and disease emerge from feedbacks on an epidemiological network. *J. R. Soc. Interface* 10:20120656. 10.1098/rsif.2012.0656 23256187 PMC3565726

[B36] Polo-Kantola P. (2022). Sleep disturbances in pregnancy: Why and how should we manage them? *Acta Obstet. Gynecol. Scand.* 101 270–272. 10.1111/aogs.14325 35238029 PMC9564426

[B37] Radhakrishnan A. Aswathy B. Kumar V. Gulia K. (2015). Sleep deprivation during late pregnancy produces hyperactivity and increased risk-taking behavior in offspring. *Brain Res.* 1596 88–98. 10.1016/j.brainres.2014.11.021 25446439

[B38] Rahimi S. Peeri M. Azarbayjani M. Anoosheh L. Ghasemzadeh E. Khalifeh N. (2020). Long-term exercise from adolescence to adulthood reduces anxiety- and depression-like behaviors following maternal immune activation in offspring. *Physiol. Behav.* 226:113130. 10.1016/j.physbeh.2020.113130 32791182

[B39] Ren H. Li K. Min Y. Qiu B. Huang X. Luo J. (2023). *Rehmannia glutinosa* polysaccharides: Optimization of the decolorization process and antioxidant and anti-inflammatory effects in LPS-stimulated porcine intestinal epithelial cells. *Antioxidants* 12:914. 10.3390/antiox12040914 37107289 PMC10136223

[B40] Roszkowska M. Krysiak A. Majchrowicz L. Nader K. Beroun A. Michaluk P. (2022). SRF depletion in early life contributes to social interaction deficits in the adulthood. *Cell. Mol. Life Sci.* 79:278. 10.1007/s00018-022-04291-5 35505150 PMC9064851

[B41] Sedov I. Cameron E. Madigan S. Tomfohr-Madsen L. (2018). Sleep quality during pregnancy: A meta-analysis. *Sleep Med. Rev.* 38 168–176. 10.1016/j.smrv.2017.06.005 28866020

[B42] Shimizu Y. Sakata-Haga H. Saikawa Y. Hatta T. (2023). Influence of immune system abnormalities caused by maternal immune activation in the postnatal period. *Cells* 12:741. 10.3390/cells12050741 36899877 PMC10001371

[B43] Tongaonkar P. Trinh K. Ouellette A. Selsted M. (2023). Inhibition of miR-146a expression and regulation of endotoxin tolerance by rhesus theta-defensin-1. *Mediators Inflamm.* 2023:8387330. 10.1155/2023/8387330 37101596 PMC10125762

[B44] Usui N. Kobayashi H. Shimada S. (2023). Neuroinflammation and oxidative stress in the pathogenesis of autism spectrum disorder. *Int. J. Mol. Sci.* 24:5487. 10.3390/ijms24065487 36982559 PMC10049423

[B45] Vázquez-González D. Carreón-Trujillo S. Alvarez-Arellano L. Abarca-Merlin D. Domínguez-López P. Salazar-García M. (2023). A potential role for neuroinflammation in ADHD. *Adv. Exp. Med. Biol.* 1411 327–356. 10.1007/978-981-19-7376-5_15 36949317

[B46] Wang P. Wang F. Ni L. Wu P. Chen J. (2021). Targeting redox-altered plasticity to reactivate synaptic function: A novel therapeutic strategy for cognitive disorder. *Acta Pharm. Sin. B* 11 599–608. 10.1016/j.apsb.2020.11.012 33777670 PMC7982492

[B47] Wei R. Zhang Y. Li Y. Wu Q. Wang Y. Li X. (2022). Altered cognition and anxiety in adolescent offspring whose mothers underwent different-pattern maternal sleep deprivation, and cognition link to hippocampal expressions of Bdnf and Syt-1. *Front. Behav. Neurosci.* 16:1066725. 10.3389/fnbeh.2022.1066725 36570704 PMC9772274

[B48] Wood C. Alexander L. Alsiö J. Santangelo A. McIver L. Cockcroft G. (2023). Chemogenetics identifies separate area 25 brain circuits involved in anhedonia and anxiety in marmosets. *Sci. Transl. Med.* 15:eade1779. 10.1126/scitranslmed.ade1779 37018416 PMC7614473

[B49] Xiao L. Yan J. Feng D. Ye S. Yang T. Wei H. (2021). Critical role of TLR4 on the microglia activation induced by maternal LPS exposure leading to ASD-like behavior of offspring. *Front. Cell Dev. Biol.* 9:634837. 10.3389/fcell.2021.634837 33748121 PMC7969707

[B50] Xu D. Zhang Y. Xie B. Yao H. Yuan Y. Yuan S. (2020). The spleen mediates chronic sleep restriction-mediated enhancement of LPS-induced neuroinflammation, cognitive deficits, and anxiety-like behavior. *Aging* 12 15446–15461. 10.18632/aging.103659 32741775 PMC7467362

[B51] Yan Y. Gu Z. Li B. Guo X. Zhang Z. Zhang R. (2022). Metabonomics profile analysis in inflammation-induced preterm birth and the potential role of metabolites in regulating premature cervical ripening. *Reprod. Biol. Endocrinol.* 20:135. 10.1186/s12958-022-01008-y 36068532 PMC9446521

[B52] Zhang Y. Cheng Y. Wang Y. Wei R. Ge Y. Kong X. (2022). Environmental enrichment reverses maternal sleep deprivation-induced anxiety-like behavior and cognitive impairment in CD-1 mice. *Front. Behav. Neurosci.* 16:943900. 10.3389/fnbeh.2022.943900 35910680 PMC9326347

[B53] Zhang Y. Wei R. Ni M. Wu Q. Li Y. Ge Y. (2023a). An enriched environment improves maternal sleep deprivation-induced cognitive deficits and synaptic plasticity via hippocampal histone acetylation. *Brain Behav.* 13:e3018. 10.1002/brb3.3018 37073496 PMC10275536

[B54] Zhang Y. Wei R. Li X. Feng Y. Zhang K. Ge Y. (2023b). Long-term environmental enrichment overcomes depression, learning, and memory impairment in elderly CD-1 mice with maternal sleep deprivation exposure. *Front. Aging Neurosci.* 15:1177250. 10.3389/fnagi.2023.1177250 37168717 PMC10164971

[B55] Zhang Z. Zeng L. Chen J. Wu Y. Wang Y. Xia L. (2022). Long-term environmental enrichment relieves dysfunctional cognition and synaptic protein levels induced by prenatal inflammation in older CD-1 mice. *Neural Plast.* 2022:1483101. 10.1155/2022/1483101 35574247 PMC9106518

[B56] Zhao Q. Peng C. Wu X. Chen Y. Wang C. You Z. (2014). Maternal sleep deprivation inhibits hippocampal neurogenesis associated with inflammatory response in young offspring rats. *Neurobiol. Dis.* 68 57–65. 10.1016/j.nbd.2014.04.008 24769004

[B57] Zhuang Z. Zhang Z. Zhang Y. Ge H. Sun S. Zhang P. (2021). A long-term enriched environment ameliorates the accelerated age-related memory impairment induced by gestational administration of lipopolysaccharide: Role of plastic mitochondrial quality control. *Front. Cell Neurosci.* 14:559182. 10.3389/fncel.2020.559182 33613195 PMC7886998

[B58] Zou W. Feng R. Yang Y. (2018). Changes in the serum levels of inflammatory cytokines in antidepressant drug-naïve patients with major depression. *PLoS One* 13:e0197267. 10.1371/journal.pone.0197267 29856741 PMC5983476

